# Molecular MR-Imaging for Noninvasive Quantification of the Anti-Inflammatory Effect of Targeting Interleukin-1β in a Mouse Model of Aortic Aneurysm

**DOI:** 10.1177/1536012120961875

**Published:** 2020-11-20

**Authors:** Julia Brangsch, Carolin Reimann, Jan Ole Kaufmann, Lisa Christine Adams, David Onthank, Christa Thöne-Reineke, Simon Robinson, Marco Wilke, Michael Weller, Rebecca Buchholz, Uwe Karst, Rene Botnar, Bernd Hamm, Marcus Richard Makowski

**Affiliations:** 1 Department of Radiology, 14903 Charité–Universitätsmedizin Berlin, Freie Universität Berlin, Humboldt-Universität zu Berlin, Berlin Institute of Health, Berlin, Germany; 2 Department of Veterinary Medicine, Institute of Animal Welfare, Animal Behavior and Laboratory Animal Science, Freie Universität Berlin, Berlin, Germany; 3 Division 1.5 Protein Analysis, Federal Institute for Materials Research and Testing (BAM), Berlin, Germany; 4 Department of Chemistry, Humboldt-Universität zu Berlin, Berlin, Germany; 5 128865 Lantheus Medical Imaging, North Billerica, MA, USA; 6 Institute of Inorganic and Analytical Chemistry, 9185 Westfälische Wilhelms-Universität Münster, Münster, Germany; 7 School of Biomedical Engineering and Imaging Sciences, 4616 King’s College London, St Thomas’ Hospital, London, United Kingdom; 8 Wellcome Trust/EPSRC Centre for Medical Engineering, 4616 King’s College London, United Kingdom; 9 BHF Centre of Excellence, 4616 King’s College London, Denmark Hill Campus, London, United Kingdom; 10 Escuela de Ingeniería, Pontificia Universidad Católica de Chile, Santiago, Chile; 11 Department of Diagnostic and Interventional Radiology, Klinikum rechts der Isar, Technical University Munich, Munich, Germany

**Keywords:** animal models of disease, cardiovascular

## Abstract

**Background:**

Molecular-MRI is a promising imaging modality for the assessment of abdominal aortic aneurysms (AAAs). Interleukin-1β (IL-1β) represents a new therapeutic tool for AAA-treatment, since pro-inflammatory cytokines are key-mediators of inflammation. This study investigates the potential of molecular-MRI to evaluate therapeutic effects of an anti-IL-1β-therapy on AAA-formation in a mouse-model.

**Methods:**

Osmotic-minipumps were implanted in apolipoprotein-deficient-mice (N = 27). One group (Ang-II+01BSUR group, n = 9) was infused with angiotensin-II (Ang-II) for 4 weeks and received an anti-murine IL-1β-antibody (01BSUR) 3 times. One group (Ang-II-group, n = 9) was infused with Ang-II for 4 weeks but received no treatment. Control-group (n = 9) was infused with saline and received no treatment. MR-imaging was performed using an elastin-specific gadolinium-based-probe (0.2 mmol/kg).

**Results:**

Mice of the Ang-II+01BSUR-group showed a lower aortic-diameter compared to mice of the Ang-II-group and control mice (p < 0.05). Using the elastin-specific-probe, a significant decrease in elastin-destruction was observed in mice of the Ang-II+01BSUR-group. In vivo MR-measurements correlated well with histopathology (y = 0.34x-13.81, R^2^ = 0.84, p < 0.05), ICP-MS (y = 0.02x+2.39; R^2^ = 0.81, p < 0.05) and LA-ICP-MS. Immunofluorescence and western-blotting confirmed a reduced IL-1β-expression.

**Conclusions:**

Molecular-MRI enables the early visualization and quantification of the anti-inflammatory-effects of an IL-1β-inhibitor in a mouse-model of AAAs. Responders and non-responders could be identified early after the initiation of the therapy using molecular-MRI.

## Introduction

Abdominal aortic aneurysms (AAAs) affect 3-4% of men over the age of 65 and account annually for over 15,000 deaths.^1^ If unrecognized or untreated, the progressive dilation of the aortic wall can lead to AAA rupture. Therefore, a high clinical need for noninvasive medical therapies of AAAs exists. The inhibition of progressive aortic dilation via a pharmacological approach could represent a substantial advancement for patients, especially as invasive therapy or surgery can be associated with severe complications. As antibody-based therapies are associated with substantial costs it would be beneficial to differentiate therapy responders from non-responders early after the initiation of the therapy.

Although the initiating events are not fully elucidated yet, inflammation was shown to be a major contributing factor to AAA formation and progression.^2^ AAA formation is characterized by proinflammatory cell infiltration, including monocytes and macrophages, the release of cytokines and proteases and breakdown of extracellular matrix (ECM) proteins.^2-5^ Especially, the destruction of medial elastin and collagen (type I and III) causes an ongoing reduction of the stability of the aortic wall leading to an increasing inability to withstand the high intraluminal pressure.^6,7^

Interleukin-1β (IL-1β) represents a gatekeeper for inflammation and contributes substantially to the progressive destruction of aortic ECM proteins during AAA development.^8^ Different preclinical and clinical studies demonstrated significantly increased levels of IL-1β in AAAs compared to healthy aortas. Therefore, the disruption of the inflammatory pathway via IL-1β neutralization represents a promising novel therapeutic target for the treatments of AAAs.^9-12^

In clinical practice, diagnosis and monitoring of AAAs include computed tomography (CT), magnetic resonance imaging (MRI) or ultrasound. Currently, there is no established biomarker available for the characterization of aortic aneurysms since the aortic diameter alone was shown to be not a reliable diagnostic and prognostic parameter for AAAs.^13,14^ Molecular MRI using probes that visualize extracellular-matrix proteins for the assessment of AAAs presents a promising new approach for aneurysm diagnosis and monitoring.^15-17^ Elastin-specific molecular MRI allows an in vivo visualization and quantification of changes in elastin composition of the aortic wall and offers, therefore, the potential for noninvasive characterization of AAAs.^14,18,19^

In this study, we investigated the feasibility of molecular MRI for the early visualization and quantification of the effects of the interleukin-1β inhibitor 01BSUR in a mouse model of AAAs with the overall aim to differentiate responders from non-responders early after the initiation of the therapy.

## Methods

### Animal Experiments

**Figure 1.**
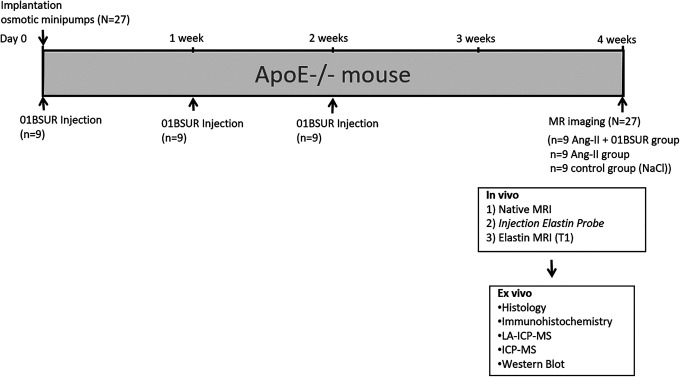
Experimental setup. Nine mice (n = 9, Ang-II + 01BSUR group) were injected subcutaneously (s.c.) with 0.3 mg of 01BSUR 3 times: on the day of minipump implantation (day 0), 7 days and 14 days after minipump implantation. The angiotensin-II group (n = 9) and the control group received no treatment after minipump implantation. MR imaging with a clinical dose of an elastin-specific gadolinium-based (0.2 mmol/kg) of all 3 groups was performed 28 days following minipump implantation.

Eight-week-old male ApoE-knockout (B6.129P2-ApoE^tm1Unc^ /J) mice were obtained from the Research Institute of Experimental Medicine at the Charité Berlin. Three groups (N = 27) of ApoE-/- mice underwent subcutaneous implantation of osmotic minipumps (Alzet model 2004, Durect Corp). To determine the effect of the interleukin-1β on AAA progression, 9 mice (n = 9, Ang-II + 01BSUR group) were continuously infused with angiotensin-II with a rate of 1000 (ng/kg)/min for 28 days and were injected subcutaneously (s.c.) with 0.3 mg 01BSUR (Novartis, Basel, Switzerland) 3 times: on the day of minipump implantation (day 0), 7 days and 14 days after minipump implantation (Figure 1). In addition, one group of mice (Ang-II group, n = 9) was also continuously infused with angiotensin-II for 28 days but received no treatment after minipump implantation. A control group of 9 mice (n = 9) received saline instead of AngII via the osmotic minipump for 28 days and received also no treatment. For MR imaging, animals were anaesthetized by an intraperitoneal injection of a combination of Medetomidine (500 µg/kg), Fentanyl (50 µg/kg), and Midazolam (5 mg/kg). MRI was then performed prior to and following the administration of 0.2 mmol/kg of an elastin-specific contrast agent (ESMA, Lantheus Medical Imaging, North Billerica, Massachusetts, USA). For ex vivo analysis, mice were sacrificed and exsanguinated by arterial perfusion of saline and the aorta including the right renal artery and the last pair of intercostal arteries was excised.

All procedures were performed according to the guidelines and regulations of the Federation of Laboratory Animal Science Associations (FELASA) and the local Guidelines and Provisions for Implementation of the Animal Welfare Act.

### Interleukin-1β Inhibitor 01BSUR

The Ang-II + 01BSUR group (n = 9) received 01BSUR (Novartis, Basel, Switzerland), an interleukin-1β inhibitor. This IgG2a/k monoclonal mouse antibody highly binds to IL-1β (dissociation constant (K_D_) = 302 pM) and shows a half-life of 14 days.^9^ By neutralizing the cytokine IL-1β, this antibody may reduce the pro-inflammatory and tissue-degrading activities of IL-1β resulting in a reduced development and progression of a variety of acute and chronic inflammatory diseases.^20^ 01BSUR has already been introduced in several preclinical studies in the context of aortic aneurysms^9^ as well as aortic calcification^21^ or collagen-induced arthritis^20^ using different rodent models.

Mice were treated 3 times with 0.3 mg (0.9 mg per mice in total) 01BSUR starting on the day of minipump implantation (day 0) followed by injections (s.c.) after 7 and 14 days. The used dose in this study is equivalent to the clinically approved dose of canakinumab used in the CANTOS study.^22^

### Gadolinium-Based Elastin-Specific Contrast Agent

The gadolinium-based elastin-specific contrast agent (ESMA, Lantheus Medical Imaging, North Billerica, Massachusetts, USA) has a small molecular weight of 856 g/mol. Showing a short blood half-life and the highest uptake within the aorta 30 to 45 minutes following the administration (13.2 ± 2.3% ID g^−1^) the agent is then predominant cleared by the renal system.^23,24^ A longitudinal relaxivity of 8.65 ± 0.42 mM^−1^ s^−1^ at 3 Tesla was reported for the agent bound to mice aortas.^23^ Due to the small molecular weight, short blood half-life, renal clearing and relaxivity, the elastin-specific contrast agent used in this study is comparable to other clinically used gadolinium-based contrast agents, including gadobuterol (Gadovist®, Bayer AG, Leverkusen Germany) and gadofosveset (Vasovist®, Bayer AG, Leverkusen Germany). The elastin specific probe has already been validated and used for the visualization and characterization of elastin in the context of atherosclerosis^23,24^ and aortic aneurysms.^18,19,25^ In this study, a clinical dose of 0.2 mmol/kg was used.

### In Vivo MR Experiments Including MR Angiography

All animals (N = 27) were imaged 4 weeks after osmotic minipump implantation (Figure 1). A clinical 3T Siemens system (Biograph, Siemens Healthcare Solutions, Erlangen, Germany) equipped with a clinical single loop coil (47 mm, Siemens Healthcare Solutions, Erlangen, Germany) was used for the imaging session. Following anesthesia injection, venous access for the administration of the contrast agent into the tail vein was established using a small diameter tube with an attached 30G cannula. Animals were then positioned in a prone position on the microscope (47 mm in diameter) single loop coil. The body temperature (37°C) of the mice was monitored using an MR-compatible heating system (Model 1025, SA Instruments Inc, Stony Brook, NY) to avoid rapid cooling.

The elastin imaging was performed as described previously.^14^ For an anatomical overview and localization of the abdominal aorta, low-resolution 3-dimensional gradient echo scout scan was performed followed by an arterial 2D TOF angiography (2-dimensional time-of-flight angiography) in transverse orientation for specific visualization of the aorta. Both sequences were performed using the following imaging parameters: Imaging matrix = 960, field of view = 200 × 200 mm, slice thickness = 0.35 mm, in plane spatial resolution = 0.166 mm (reconstructed 0.15 mm), flip angle = 90°, repetition time (TR) sequence = 35 ms and echo time (TE) 4,4 ms. From the arterial time-of-flight dataset, a maximum intensity projection (MIP) was generated for display of an arterial angiogram of the abdominal aorta and abdominal aortic aneurysms, to plan the subsequent contrast-enhanced sequences. The inversion recovery scan to visualize the gadolinium-based contrast agent was preceded by a 2D TI scout sequence planned perpendicular to the abdominal aorta, which was used to determine the optimal inversion time (TI) for blood signal nulling. Imaging parameters of the TI scout sequence included: FOV = 180 mm, matrix = 750, in plane spatial resolution = 0.24 × 0.24 mm, slice thickness = 1 mm, TR between subsequent IR pulses = 1000 ms, and flip angle = 15°. Imaging parameters of the high-resolution 3D inversion FLASH sequence scan employed for visualization of gadolinium-based molecular probe were: FOV = 50 mm, matrix = 416, in plane spatial resolution = 0.12 × 0.12 mm, slice thickness = 0.3 mm, slices = 56, TR/TE = 10.1/7.0 ms, TR between subsequent IR pulses = 1000 ms, and flip angle = 30°.

### Assessment of Magnetic Resonance Imaging Signal

Morphometric measurements were conducted on high–resolution MRI images using OsiriX (version 7.1, OsiriX foundation) as previously described.^14^ Regions of interests (ROIs) were defined as areas of signal enhancement, which were co-localized with areas of aneurysmal aortic tissue. For these areas, the contrast-to-noise-ratio (CNR) was calculated as follows: CNR = (Combined vessel wall and aneurysmal aortic tissue signal—blood signal) / standard deviation signal (noise). Noise was defined as the standard deviation in pixel intensity from a ROI placed in the background air anterior to the aorta.

### Histological Analysis of Aortic Aneurysms and Aortic Aneurysm Morphometry

For histological analysis, aortic tissue was processed overnight in MorFFFix® (Morphisto, Frankfurt am Main, Germany). The aortas were embedded in paraffin and cut into 5 µm thick serial sections. Miller’s Elastica van Gieson stain (EvG) was performed for visualization of elastic fibers. Additionally, Hematoxylin and Eosin (HE) staining was performed. For morphological measurements, histological slices were photographed using a light microscope (Keyence BzX800) and analyzed using computer-assisted image analysis (ImageJ software, Version 1.51) to determine the %EvG stain area per adventitial area as previously described.^14,18^

### Immunofluorescence Analysis

Immunofluorescence staining was performed to visualize interleukin-1β within the aortic wall. After embedding in OCT compound, abdominal aortic tissues were cut at −20 °C into 10 µm thick serial cryosections. Sections were treated with polyclonal IL-1β antibody (Rabbit anti-Mouse IL-1 beta, Bio-Rad 1:100, 1 hour at room temperature) followed by a polyclonal secondary antibody AlexaFluor 568 (Donkey anti Rabbit IgG, Thermo Fisher Scientific, Germany, 1:200, 1 hour at room temperature) each diluted in Dako REAL^TM^ Antibody Diluent (Dako, Denmark). Counterstaining and mounting were performed using Roti^®^-Mount FluorCare (Carl Roth, Germany).

### Western Immunoblot Analysis

To evaluate the levels of IL-1β and CD68 in the aortic wall of the Ang-II + 01BSUR group and Ang-II group, Western immunoblot analysis was performed. Aortic tissues (n = 3 per group) were homogenized and solubilized in RIPA buffer (pH = 7.4). The protein concentration was determined using UV-spectrometry at 280 nm (Evolution 220 UV-Visible Spectrometer, Thermo Scientific^TM^). 5 mg/ml total protein per lane diluted in SDS loading buffer was loaded onto 4-12% Tris-Glycine Gels (ServaGel TG Prime Vertical Tris-Glycine Gel 4-12%). Proteins were separated by SDS-PAGE (Hoefer SE260). To assure equal amounts of total protein loaded per lane, GAPDH was used as loading control. Due to the identical molecular weights of the targeted proteins CD68 and GAPDH, 2 gels were run simultaneously. Proteins were transferred to PVDF membranes using Trans-Blot Turbo RTA transfer kit (Bio-Rad). Primary antibodies (Rabbit anti-Mouse IL-1β, Bio-Rad 1:500; Rabbit anti-Mouse CD68, antibodies.com, 1:500; Rabbit anti-Mouse GAPDH, Invitrogen 1:1000) were detected using a secondary HRP-labeled antibody (Goat anti-Rabbit IgG (H/L): HRP, Bio-Rad 1:1000) and SeramunBlau® blotting substrate (Seramun Diagnostica GmbH). Protein bands were quantified by ImageJ (Version 1.51).

### Inductively Coupled Mass Spectroscopy (ICP-MS)

For a reproducible measurement of the local gadolinium concentration, ICP-MS was performed. Aortic tissue samples (n = 3 per group) were digested overnight in 70% nitric acid at 37°C. For ICP-MS analysis samples were diluted with deionized water. A standard curve was acquired for the concentration of gadolinium for each analysis.

### Element Specific Bioimaging Using Laser Ablation (LA) Coupled to Inductively Coupled Plasma-Mass Spectrometry (LA-ICP-MS)

LA-ICP-MS was performed as described previously.^14^ Aneurysms were cut into 10 µm cryosections at −20°C and mounted on SuperFrost Plus adhesion slides (Thermo Scientific). The LA-ICP-MS analysis was performed using an LSX 213 G2+ laser system (CETAC Technologies, Omaha, USA) which was equipped with a 2-volume HelEx II cell connected via Tygon tubing to an ICPMS-2030 (Shimadzu, Kyoto, Japan). With a spot size of 10 µm samples were ablated via line-by-line scan using a scan speed of 30 µm/s and 800 mL/min He as transport gas. The analysis was performed in collision gas mode with He as collision gas, with 50 ms integration time for the analyzed isotopes ^31^P, ^57^Fe, and ^64^Zn and 75 ms integration time for the 2 Gd isotopes ^158^Gd and ^160^Gd. Matrix-matched standards based on gelatin were used for the quantification of Gd. Nine gelatin standards (10% w/w) including a blank, were spiked with different Gd concentrations (1 to 500 µg/g). Averaged intensities of the scanned lines of the standards were in good linear correlation with a regression coefficient R^2^ = 0.998 within this concentration range. Limit of detection (LOD) and limit of quantification (LOQ), calculated with the 3σ- and 10σ-criteria, were 16 ng/g and 54 ng/g Gd. The quantification and visualization were performed using in-house developed software (WWU Münster, Münster, Germany).

### Statistical Analysis

Values are specified as mean ± standard deviation. An unpaired, 2-tailed Student’s t-test was applied for the comparison of continuous variables. Linear regression was applied to determine the relationship between in vivo and the ex vivo measurements. P-value < 0.05 was considered to indicate a statistically significant difference.

## Results

Overall 27 mice (N = 27) were included until the end point of the study. Figure 1 summarizes the detailed study setup including the different groups of mice.

### MR Angiographic Measurements for the Assessment of the Area of the Aortic Lumen

**Figure 2.**
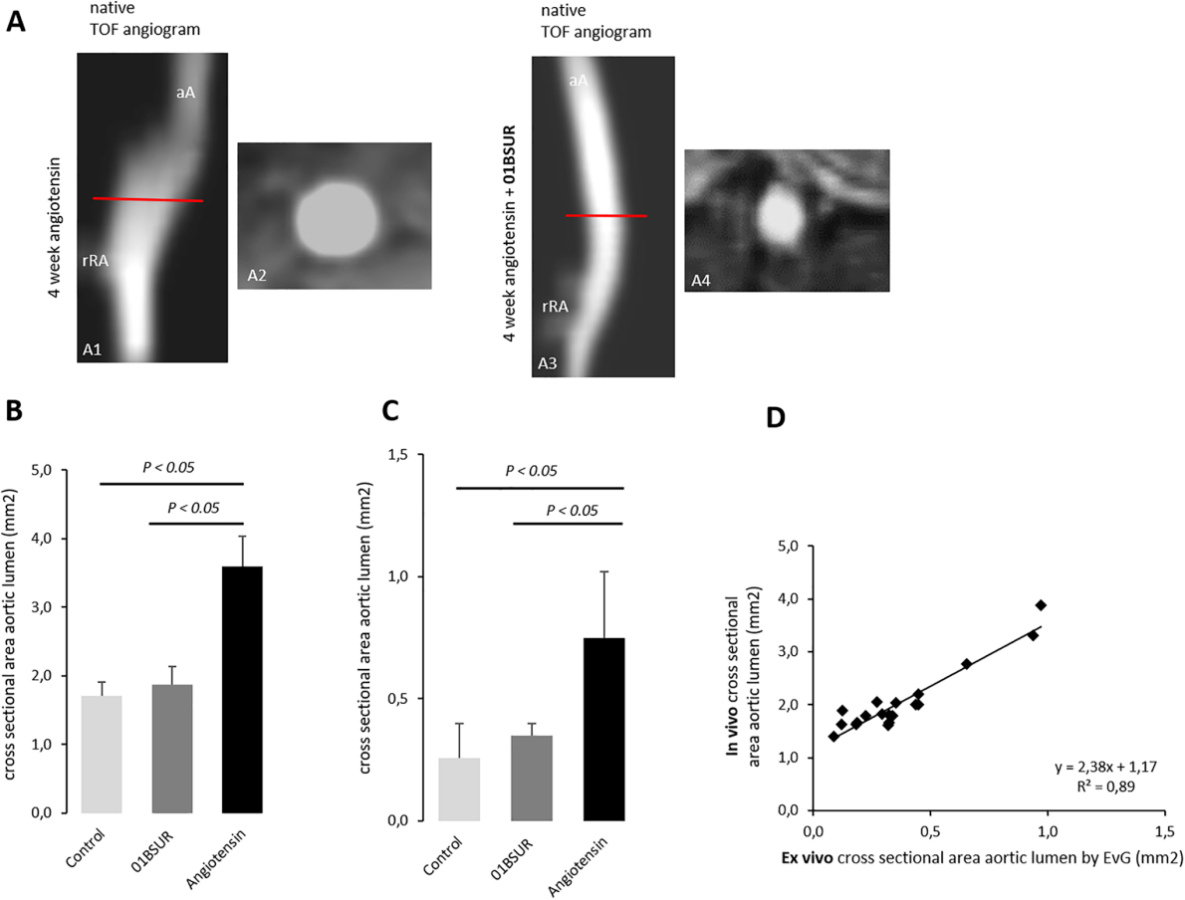
In vivo and ex vivo assessment of the aortic cross-sectional area. The Time-of-flight (TOF) angiogram of the suprarenal part of the abdominal aorta 4 weeks after continuous infusion of angiotensin II showed a strong dilatation of the aortic lumen (A1, A2) whereas a non-dilated aortic lumen was observed in mice treated with 01BSUR (A3, A4). On in vivo cross-sectional area measurements (B) and ex vivo histological measurements (C) a significant increase in cross-sectional areas of the aortic lumen size was observed in untreated mice after 4 weeks of angiotensin II infusion, reflecting development of AAAs in these mice in contrast to mice treated with 01BSUR. There was no significant difference in the cross-sectional area of the aortic lumen size between the Ang-II + 01BSUR group and the control group. In vivo MR measurements of the aortic diameter correlated strongly with ex vivo measurements on histological Elastica van Gieson (EvG) stained sections (D). aA: suprarenal abdominal-aorta; rRA: right renal-artery.

The angiography of the mice of the control group (ApoE-/- mice after 4 weeks of saline infusion, n = 9) showed a non-dilated aortic lumen. The continuous Ang-II infusion in mice (n = 9) resulted in a significantly enlarged aortic lumen and dilatation of the aortic wall in the suprarenal region (Figure 2A). AngII-infused mice treated 3 times with 01BSUR (n = 9) showed a substantially lower aortic diameter in vivo and ex vivo compared to mice without 01BSUR treatment (p < 0.05) (Figure 2B, C). No significant difference in aortic diameter could be observed in vivo and ex vivo between the control group and Ang-II + 01BSUR group (p > 0.05) (Figure 2B, C). In vivo measurements were in good agreement with ex vivo measurements of the cross-sectional area on histological sections (y = 2.38x−1.17, R^2^ = 0.89; p < 0.05, Figure 2D).

### Molecular MRI for the Noninvasive Assessment of the Effects of IL-1β Inhibitor 01BSUR on AAA Formation

**Figure 3.**
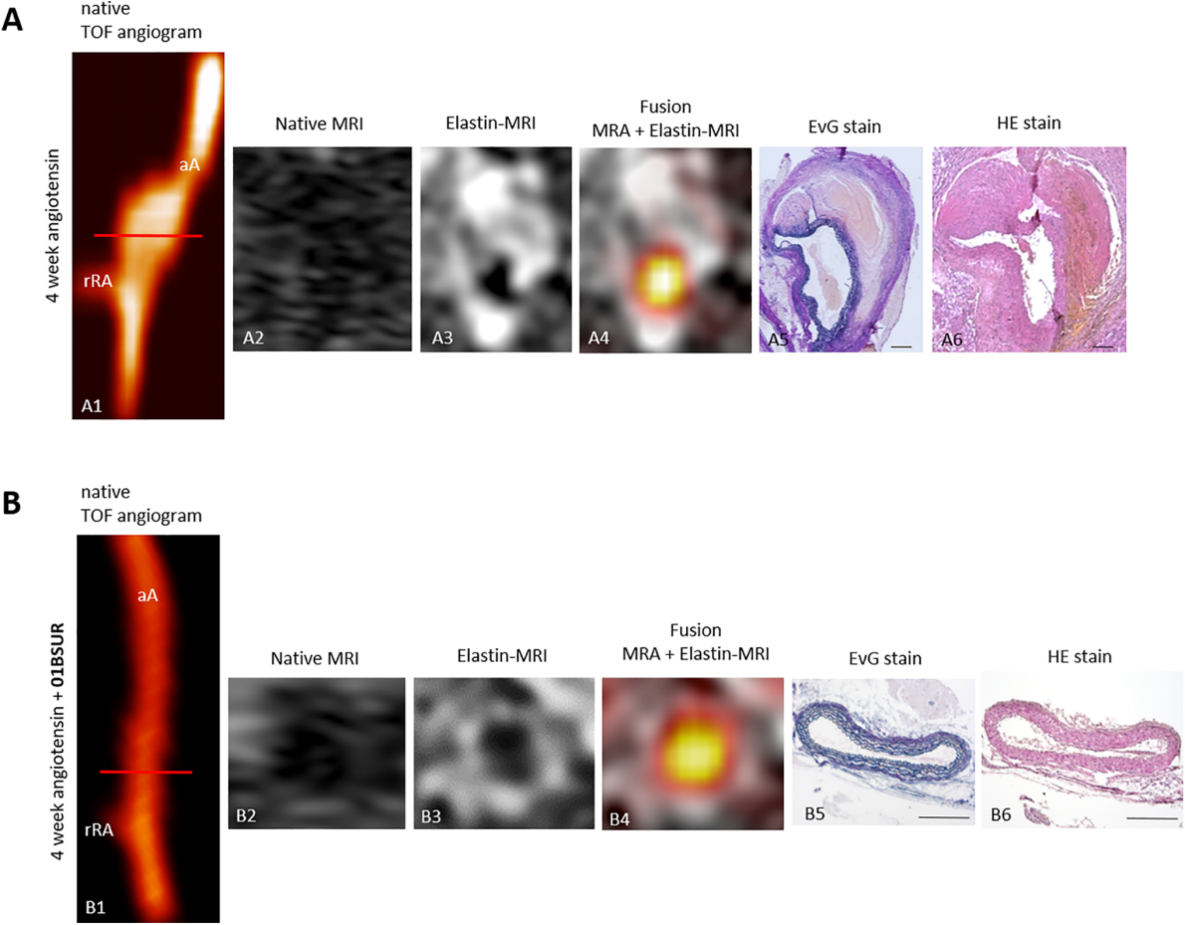
In vivo molecular MRI of extracellular-matrix of the aortic wall. Time-of-flight angiograms showing a suprarenal abdominal aorta including the right renal-artery of a male ApoE-/- mouse after 4 weeks of Ang-II infusion (A1). An extensive aortic aneurysm was developed after 4 weeks of Ang-II infusion including a strong remodeling of the extracellular matrix by an expression of elastic-fibers in the area of former elastin dissection which was observed *in vivo* by MRI after the administration of the elastin-specific-probe (A3, A4) and *ex vivo* by histological analysis (A5, A6). The abdominal aorta of a male ApoE-/- mouse treated with 01BSUR show no pathological changes of the aortic wall *in vivo* on the time-of-flight angiogram (B1), native MRI (B2) and T1-weighted-sequences using the elastin-specific-probe (B2, B3) or on corresponding *ex vivo* histology (B5, B6). Scale bars represent 200 µm. TOF: Time-of-flight, EvG: Elastica van Gieson staining, Elastic fibers are stained blue-black; HE: Hematoxylin-Eosin-staining; MRA: magnetic-resonance-angiography; aA: suprarenal abdominal-aorta; rRA: right renal-artery.

ApoE-/- mice (n = 9) developed extensive aortic aneurysm after 4 weeks of AngII-infusion as described previously including infiltration of inflammatory cells and ECM remodeling.^14^ Using the elastin-specific probe, a strong expression of elastin within the aortic wall could be observed in vivo in these mice due to a strong remodeling of the extracellular matrix (ECM) especially in the areas of former elastic fiber dissection. In contrast, 01BSUR treatment reduced the impact of Ang-II on elastin levels, prevent the destruction of elastic fibers within the aortic wall and therefore the development of abdominal aortic aneurysms (Figure 3). The control group receiving saline, showed no pathological changes of the aortic wall.

### T1-Weighted MR Imaging for the Assessment of the Gadolinium-Based Elastin-Specific Probe

**Figure 4.**
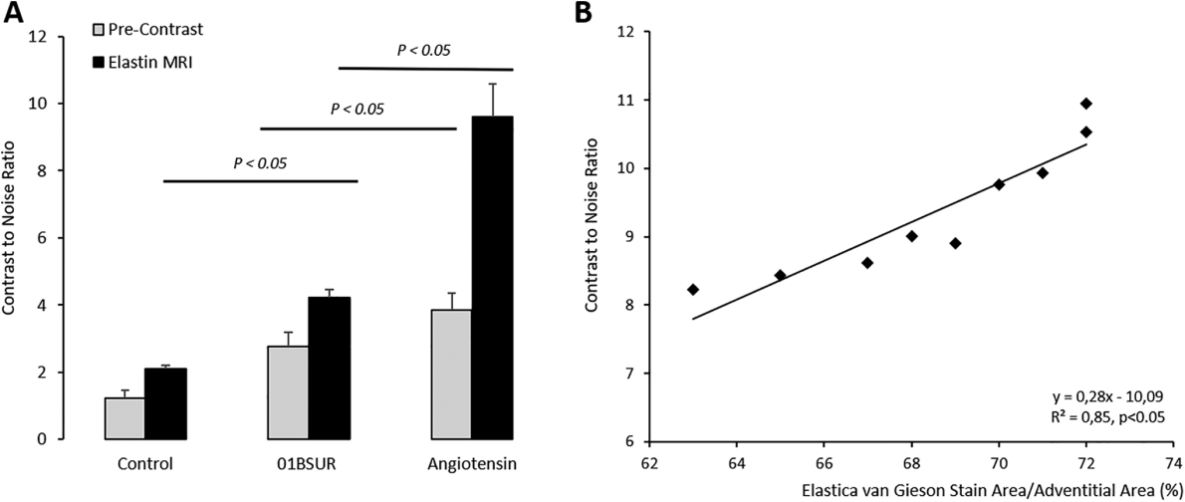
In vivo MRI signal measurements and ex vivo quantification of the gadolinium-based elastin specific probe. Contrast-to-noise-ratio (CNR) values before and following the administration of the gadolinium-based elastin-specific MR probe showed a significant increase in CNR in the aortic wall in mice of the Ang-II + 01BSUR group, Ang-II group and control group. The strongest signal enhancement was shown by the mice of the Ang-II group due to a strong remodeling and expression of elastic fibers in the aneurysmal wall. In vivo CNR measurements showed a strong correlation with ex vivo Elastica van Gieson (EvG) staining on corresponding histological sections (B).

Prior to the administration of the elastin-specific MR probe, a low contrast-to-noise-ratio was measured in the aortic wall of all 3 groups. A significant (p < 0.05) increase in CNR in the aortic wall was measured for mice receiving Ang-II, Ang-II + 01BSUR and the control mice following the administration of the elastin-specific agent (Figure 4A). The Ang-II group showed a strong signal enhancement in the area of the aneurysmal wall due to extracellular matrix remodeling by expression of elastic fibers in the area of former disruption of the internal elastic lamina. The Ang-II + 01BSUR group showed a significantly weaker signal enhancement in the aortic wall indicating an absence of aortic rupture and prevention of AAA formation. In vivo measurements correlated with ex vivo histological measurements of elastic fiber density using Elastica van Gieson staining (y = 0.34x−13.81, R^2^ = 0.85; p < 0.05, Figure 4B).

### Correlation of In Vivo Measurements of the Elastin Specific Probe With Ex Vivo Histology

For ex vivo measurements on histological sections, the Elastica van Giesson (EvG) staining was used to assess the amount and distribution of elastic fibers in the aortic wall (Figure 3A4, B4). The Ang-II group showed the development of aortic aneurysms due to the dissection of internal elastic fibers. After 4 weeks of AngII-infusion a strong remodeling of the aortic wall was observed at these areas. Due to this compensatory repair process, a significant increase in elastic fibers in both, in vivo and ex vivo measurements was observed in mice of the AngII-group. A good correlation was shown between ex vivo histological measurements and in vivo measurements using the elastin-specific probe (y = 0.34x−13.81 R^2^ = 0.85; Figure 4B).

### Expression of IL-1β in the Aortic Wall by Immunofluorescence and Western Blot

**Figure 5.**
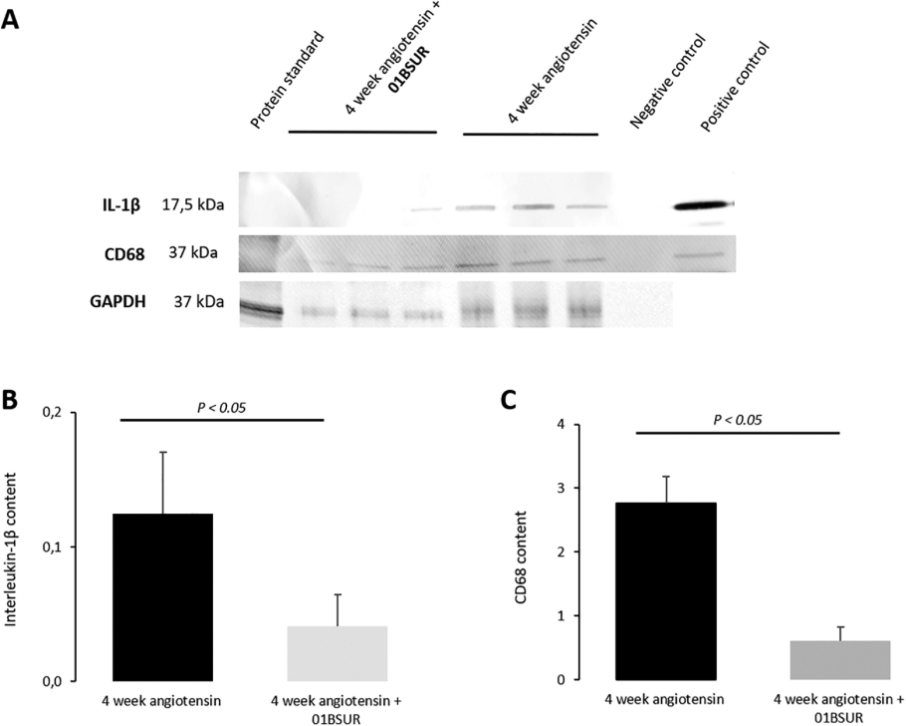
Western Blot analysis. Western Blot analysis (A) and quantification of protein bands (B, C) of suprarenal aortic tissue of ApoE-/- mice after 4 weeks of angiotensin II infusion (n = 3) and ApoE-/- mice after 4 weeks of angiotensin II infusion treated with 01BSUR (n = 3) using an interleukin-1β (17,5 kDa) antibody and CD68 (37 kDa) antibody. GAPDH (37 kDa) was used as loading control. A cropped Blot is displayed to improve conciseness.

**Figure 6.**
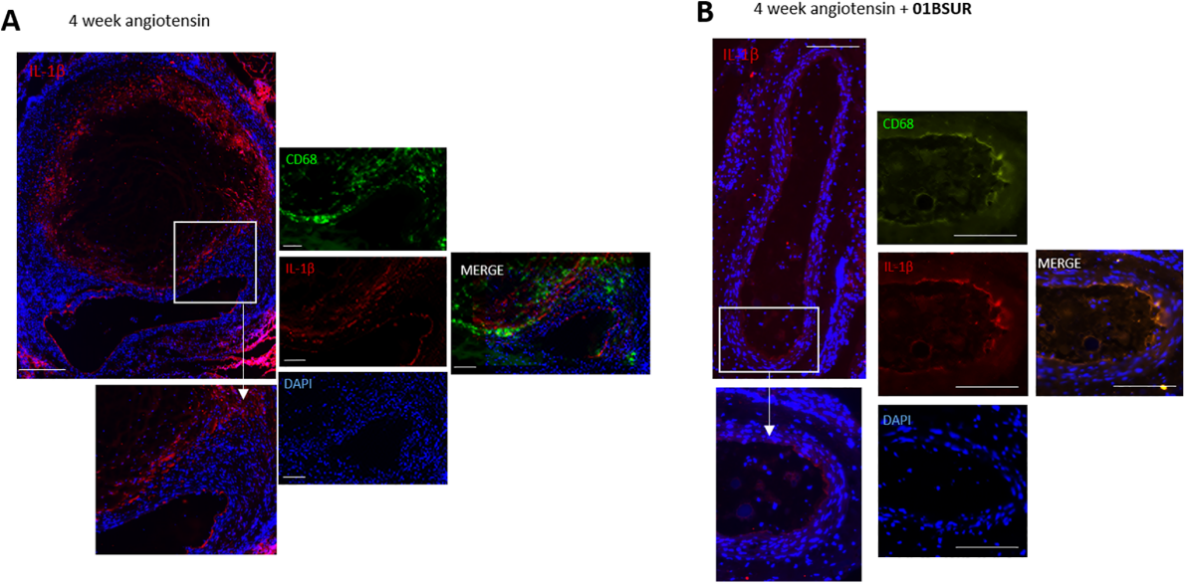
Ex vivo immunofluorescence measurements. Immunofluorescence staining with CD68 antibody was performed on histological slices of mice of the Ang-II group (A) and Ang-II + 01BSUR group (B). **A:** Mice of the AngII-group showed an increased expression of IL-1β in the aortic wall (on the left hand; scale bar represent 200 µm). On the right hand: Macrophages (CD68) are shown in green, Interleukin-1β in red and cell nuclei (DAPI) in blue. The stacked image demonstrates a clear co-localization of IL-1β with macrophages. Scale bars represent 100 µm. **B**: Mice of the Ang-II + 01BSUR group showed a significant lower IL-1β expression (on the left hand) as well as a trivial macrophage staining (on the right hand). Scale bars represent 200 µm. Different magnifications were used due to the relatively large size of the aneurysm.

For the evaluation of the expression of IL-1β within the aortic wall, Western Blot analysis and immunofluorescence staining of histological sections were performed. Western Blot analysis of the abdominal aorta (n = 3 per group) showed a significantly lower expression of IL-1β within the aortic wall in mice treated with 01BSUR compared with the AngII group (Figure 5A, B). These results were confirmed by histological analysis which also showed a significant decrease in IL-1β expression around the aorta after 01BSUR treatment (Figure 6).

In addition, to evaluate the effects of 01BSUR on macrophage recruitment and accumulation, Western Blot analysis and immunofluorescence CD68 staining of adjacent histological slices were performed. Mice of the AngII group demonstrated extensive macrophage staining and a significant higher expression of CD68 whereas mice treated with 01BSUR had trivial macrophage staining (Figure 6) and lower CD68 expression in Western Blot analysis (Figure 5A, C). Furthermore, immunofluorescence microscopy showed that IL-1β clearly co-localized with macrophages within the aortic wall (Figure 6). These findings are consistent with 01BSUR suppressing inflammatory processes in aneurysm development.

### Spatial Localization of the Gadolinium-Based Elastin-Specific Probe Using Laser Coupled Mass Spectrometry (LA-ICP-MS)

**Figure 7.**
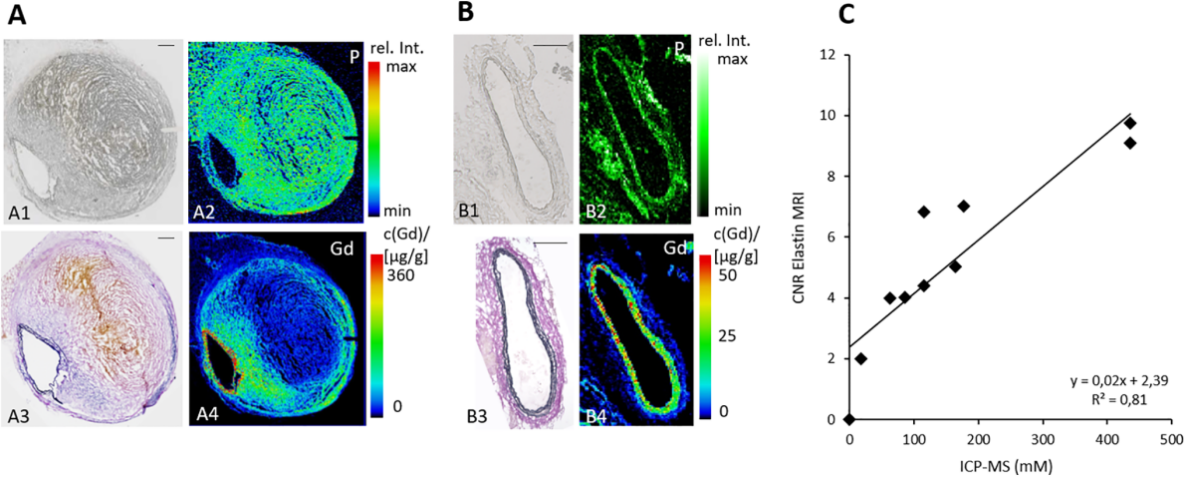
Spatial localization of the elastin-specific gadolinium-based probe in the aortic wall using laser coupled mass spectrometry (LA-ICP-MS) and correlation of *in vivo* MRI signal measurements with ICP-MS. The phosphorus distribution was measured using LA-ICP-MS to get an anatomical overview of the histological sections (A2, B2). LA-ICP-MS visualized the elastin-specific gadolinium-based probe within the aneurysmal wall (A4, B4) by the Gd signal. A clear co-localization of gadolinium accumulation with the elastic fibers in the Elastica van Gieson stain was shown (A3, B3). Scale bars represent 200 µm. C: A strong correlation of ICP-MS measurements for gadolinium with *in vivo* CNR measurements following the administration of the elastin-specific probe was demonstrated. CNR: Contrast to noise ratio; ICP-MS: Inductively coupled mass spectroscopy.

LA-ICP-MS was performed to visualize the spatial distribution of gadolinium within the aortic wall. Sections of the aortic wall from mice of the Ang-II + 01BSUR group (n = 3) and AngII group (n = 3) after 4 weeks of AngII-infusion showed a strong co-localization of targeted gadolinium with elastic fibers (Figure 7A, B).

### Gadolinium Concentration by Inductively-Coupled Mass Spectrometry (ICP-MS)

For determination of the concentration of gadolinium from the elastin-specific contrast agent in the aortic wall, inductively coupled mass spectrometry (ICP-MS) was performed in 9 mice (n = 3 of each group). A strong correlation of in vivo CNR measurements and ex vivo measured gadolinium concentrations by ICP-MS was shown (y = 0.02x + 2.39; R^2^ = 0.81, p < 0.05) (Figure 7C).

## Discussion

This study demonstrates the potential of molecular elastin specific MRI to evaluate the effect of disrupting IL-1β signaling on AAA formation in ApoE-/- mice. Using an elastin-specific probe, the development of an abdominal aneurysm, as well as the response to therapy, could reliably be visualized in one imaging session after 4 weeks of AngII-infusion. This non-invasive in vivo visualization and quantification by molecular MRI could improve the early evaluation of response to therapy in patients and could help to identify responders and non-responders early after initiation of the therapy.

Ex vivo histology and Western Blot analysis confirmed a reduction of inflammatory processes and IL-1β expression within the aortic wall due to the 01BSUR treatment. Further ex vivo examination by LA-ICP-MS and ICP-MS proved the specific binding of the molecular probe and correlated well with in vivo MR measurements.

### Role of IL-1β During AAA Formation

Inflammation has been shown to be a key process in AAA formation and progression including several different cytokines and proinflammatory cells. Interleukin-1β is one of the key mediators of inflammation and previous studies demonstrated its critical role in experimental aortic aneurysm development^8,10,26^ Furthermore, IL-1β was found to be elevated 4-fold in human AAAs compared to healthy aortas while circulating IL-1β is increased by nearly 10-fold in AAA patients.^11,12,27,28^ By inducing the production of matrix metalloproteinases (MMPs) in fibroblastic cells and macrophages, including MMP-9, a protease with high elastolytic activity, IL-1β contributes crucially to the progressive destruction of aortic ECM proteins during AAA formation and progression.^27,29^ Especially the loss of medial elastin fibers is thought to be the initiating event of aneurysm development.^30^ Therefore, IL-1β antagonism represents a promising approach for AAA treatment by disruption of the inflammatory pathway.

There are several pharmacological agents targeting the IL-1β pathway, including direct antibodies to IL-1β or IL-1 receptor (IL-1R). Although IL-1β neutralization was once thought to be predominantly limited to the treatment of autoimmune and chronic inflammatory diseases, including gout, rheumatoid arthritis or type 2 diabetes, the role of IL-1β in cardiovascular diseases has gained more and more in importance.^31^

Anakinra, a commercially available recombinant human IL-1R antagonist, has previously been evaluated in an experimental abdominal and thoracic aortic aneurysms model. Johnston et al. treated C57Bl/6 mice following elastase aortic perfusion with escalating doses of anakinra.^10,26^ Significant protection against AAA progression was shown in mice treated with anakinra 3 or 7 days following AAA initiation and furthermore mice treated prior to elastase exposure showed a dose-dependent decrease in maximal aortic dilation. This study indicates that IL-1β is critical for both the initiation and progression of experimental AAAs. A major clinical limitation of this study represents the application of anakinra at a dose of 100 (mg/kg)/day which exceeds the dosage approved for patient by 100-fold.

The direct inhibition of IL-1β using the monoclonal antibody 01BSUR was also recently assessed in different mouse models including Kawasaki Disease, renal inflammation and AAAs.^9,32,33^ Isoda et al. showed that treatment with 01BSUR decreased AngII-induced AAAs in IL-1 Ra-deficient mice.^9^ Histological analyses were performed after 28 days of AngII-infusion and revealed decreased degeneration of smooth muscle cells, elastic fibers and a decreased accumulation of inflammatory cells. These findings are consistent with the current study. Moreover, in our study, the effects of 01BSUR treatment were not only evaluated by ex vivo histology but more importantly by noninvasive in vivo molecular MR imaging.

### Molecular MRI for the Noninvasive Assessment of Treatment of Aortic Aneurysms

For the diagnosis and evaluation of AAAs, MRI using molecular probes for the visualization of extracellular matrix proteins has shown potential in experimental studies in the last several years. Native MR imaging enables solely the assessment of the aortic diameter which may be an unreliable marker for the risk of rupture and expansion rate and therefore the inflammatory status of aortic aneurysm.^34,35^ The gadolinium-based elastin-specific probe used in the present study allows the in vivo visualization of the elastin remodeling of the aortic wall at different stages of aortic aneurysms which has been shown by our research group^14^ and others.^18,36^ In our current study we showed that the use of this probe enables the early in vivo evaluation and quantification of AAAs and furthermore the response to the anti-inflammatory treatment with 01BSUR.

To our knowledge, this study represents the first molecular MRI study on the in vivo effects of anti-inflammatory therapy of experimental AAAs.

### Translational Potential of This Study

Currently, there are no widely accepted medical options available to reduce or slow AAA progression. The recommended treatment paradigm includes monitoring of disease progression eventually followed by surgical intervention of AAAs larger than 55 mm while the management of asymptomatic medium-sized AAAs (40-55 mm) remains still controversial.^37,38^ Therefore, there is a tremendous need for medical therapy for the treatment of patients with abdominal aneurysms.

A fully human monoclonal antibody directed against IL-1β, canakinumab, was previously presented following investigation in a large phase 3 trial for the prevention of recurrent myocardial infarction.^22^ The anti-inflammatory therapy at a dose of 150 mg every 3 months led to a significantly lower rate of recurrent cardiovascular events than placebo, independent of lipid-level lowering, in patients with previous myocardial infarction. In addition, a phase IIb trial investigating canakinumab treatment in high-risk diabetic patients demonstrated a significant dose-dependent reduction in various major inflammatory biomarkers (C-reactive protein, IL-6 and fibrinogen).^39^ These data support the potential of this novel therapeutic option for several inflammatory cardiovascular diseases, including AAAs. Regarding clinical translation in our present study, mice were treated with the clinically approved dose of an anti-IL-1β antibody as used within the CANTOS study. Using molecular MRI in addition to the evaluation of an anti-inflammatory therapy of cardiovascular diseases, responders and non-responders could be identified early after the initiation of the therapy. Such an approach could be helpful to identify the most suited patient collective, which response to treatment early after the initiation of the therapy. Additionally, this could help to identify patient collectives which would benefit from a potentially increased dose and reduce the costs as only responders would be continuously treated.

Another advantage of this study regarding clinical translation includes the use of a clinical 3 Tesla MR scanner. Since we have not used an ultrahigh field preclinical MR scanner for small animals, relaxation, rotational correlation, and signal properties can be directly translated into a clinical setting. Furthermore, the elastin-specific probe used in this study, is comparable to contrast agents already used in clinical practice, regarding molecular size and composition, as well as blood clearance and was administered at a clinical dose.^23^

### Mouse Model Used in This Study

In the small animal model used in this study, AngII-infused male apolipoprotein deficient mice develop suprarenal AAAs spontaneously without the need for surgical intervention.^40^ The formation of AAAs in this murine model and humane aneurysm formation have numerous commonalities, including medial elastic fiber degeneration, inflammatory processes and thrombus formation.^41,42^ AngII-infusion promotes macrophage infiltration, MMP activation as well as an increased release of cytokines, including IL-1β.^43,44^

### Study Limitations

The selected therapy with 01BSUR did not only reduce but prevent the formation of AAA in our current study. Therefore, a reduction in aneurysm size or inflammation within the aortic wall following AAA diagnosis with molecular MRI could not be evaluated. To further evaluate the feasibility of molecular imaging using an elastin-specific contrast agent for the monitoring of AAA therapy, a modified study design will be necessary, for example including a therapy onset after induction auf aneurysms. Consequently, more studies are needed, also to prove a possible translation into a clinical setting of AAA therapy monitoring using molecular MRI.

Another limitation of the present study represents the used mouse model. Although it closely models human AAA formation, the murine AAAs develop in the suprarenal region, whereas in humans AAAs develop preferentially in the infrarenal region.^40^ Furthermore, the elastin-specific probe used in this study is currently not approved for clinical use and a safety profile has to be investigated before translation into a patient setting.

## Conclusions

Molecular MRI enables the early visualization and quantification of the anti-inflammatory effects of the IL-1β inhibitor 01BSUR in an ApoE-/- mouse model of AAAs. Responders and non-responders might be identified early after the initiation of the therapy using molecular MRI.

## References

[1] Benjamin EJ Muntner P Alonso A, et al. Heart disease and stroke statistics—2019 update: a report from the American Heart Association. Circulation. 2019;139(10):e56–e528. doi:10.1161/CIR.0000000000000659 30700139 10.1161/CIR.0000000000000659

[2] Shimizu K Mitchell RN Libby P Inflammation and cellular immune responses in abdominal aortic aneurysms. Arterioscler Thromb Vasc Biol. 2006;26(6):987–994. doi:10.1161/01.ATV.0000214999.12921.4f 16497993 10.1161/01.ATV.0000214999.12921.4f

[3] Anidjar S Dobrin PB Eichorst M, et al. Correlation of inflammatory infiltrate with the enlargement of experimental aortic aneurysms. J Vasc Surg. 1992;16(2):139–147. doi:10.1067/mva.1992.35585 1386635 10.1067/mva.1992.35585

[4] Brophy CM Reilly JM Smith GJ, et al. The role of inflammation in nonspecific abdominal aortic aneurysm disease. Ann Vasc Surg. 1991;5(3):229–233. doi:10.1007/BF02329378 2064915 10.1007/BF02329378

[5] Freestone T Turner RJ Coady A, et al. Inflammation and matrix metalloproteinases in the enlarging abdominal aortic aneurysm. Arterioscler Thromb Vasc Biol. 1995;15(8):1145–1151. 7627708 10.1161/01.atv.15.8.1145

[6] Hellenthal FA Buurman WA Wodzig WK, et al. Biomarkers of AAA progression. Part 1: extracellular matrix degeneration. Nat Rev Cardiol. 2009;6(7):464–474. doi:10.1038/nrcardio.2009.80 19468292 10.1038/nrcardio.2009.80

[7] Farand P Garon A Plante GE Structure of large arteries: orientation of elastin in rabbit aortic internal elastic lamina and in the elastic lamellae of aortic media. Microvasc Res. 2007;73(2):95–99. 2006/12/19 doi:10.1016/j.mvr.2006.10.005 17174983 10.1016/j.mvr.2006.10.005

[8] Dinarello CA A clinical perspective of IL-1β as the gatekeeper of inflammation. Eur J Immunol. 2011;41(5):1203–1217. doi:10.1002/eji.201141550 21523780 10.1002/eji.201141550

[9] Isoda K Akita K Kitamura K, et al. Inhibition of interleukin-1 suppresses angiotensin II-induced aortic inflammation and aneurysm formation. Int J Cardiol. 2018;270:21–227. doi:10.1016/j.ijcard.2018.05.072 29884291 10.1016/j.ijcard.2018.05.072

[10] Johnston WF Salmon M Su G, et al. Genetic and pharmacologic disruption of interleukin-1β signaling inhibits experimental aortic aneurysm formation. Arterioscler Thromb Vasc Biol. 2013;33(2):294–304. doi:10.1161/ATVBAHA.112.300432 23288154 10.1161/ATVBAHA.112.300432 PMC3632435

[11] Juvonen J Surcel HM Satta J, et al. Elevated circulating levels of inflammatory cytokines in patients with abdominal aortic aneurysm. Arterioscler Thromb Vasc Biol. 1997;17:2843–2847. doi: 10.1161/01.atv.17.11.2843 9409264 10.1161/01.atv.17.11.2843

[12] Pearce WH Sweis I Yao JS, et al. Interleukin-1 beta and tumor necrosis factor-alpha release in normal and diseased human infrarenal aortas. J Vasc Surg. 1992;16(5):784–789. 1433667

[13] Richards JM Semple SI MacGillivray TJ, et al. Abdominal aortic aneurysm growth predicted by uptake of ultrasmall superparamagnetic particles of iron oxide: a pilot study. Circ Cardiovasc Imaging. 2011;4(3):274–281. doi:10.1161/CIRCIMAGING.110.959866 21304070 10.1161/CIRCIMAGING.110.959866

[14] Brangsch J Reimann C Kaufmann JO, et al. Concurrent molecular magnetic resonance imaging of inflammatory activity and extracellular matrix degradation for the prediction of aneurysm rupture. Circ Cardiovasc Imaging. 2019;12(3):e008707 doi:10.1161/CIRCIMAGING.118.008707 30871334 10.1161/CIRCIMAGING.118.008707

[15] Turner GH Olzinski AR Bernard RE, et al. Assessment of macrophage infiltration in a murine model of abdominal aortic aneurysm. J Magn Reson Imaging. 2009;30(2):455–460. doi:10.1002/jmri.21843 19629967 10.1002/jmri.21843

[16] Yao Y Wang Y Zhang Y, et al. In vivo imaging of macrophages during the early-stages of abdominal aortic aneurysm using high resolution MRI in ApoE mice. PLoS One. 2012;7(3):e33523 doi:10.1371/journal.pone.0033523 22448249 10.1371/journal.pone.0033523 PMC3308989

[17] Klink A Heynens J Herranz B, et al. In vivo characterization of a new abdominal aortic aneurysm mouse model with conventional and molecular magnetic resonance imaging. J Am Coll Cardiol. 2011;58(24):2522–2530. doi:10.1016/j.jacc.2011.09.017 22133853 10.1016/j.jacc.2011.09.017 PMC4095885

[18] Botnar RM Wiethoff AJ Ebersberger U, et al. In vivo assessment of aortic aneurysm wall integrity using elastin-specific molecular magnetic resonance imaging. Circ Cardiovasc Imaging. 2014;7(4):679–689. doi:10.1161/CIRCIMAGING.113.001131 24871347 10.1161/CIRCIMAGING.113.001131

[19] Makowski MR Preissel A von Bary C, et al. Three-dimensional imaging of the aortic vessel wall using an elastin-specific magnetic resonance contrast agent. Invest Radiol. 2012;47(7):438–444. doi:10.1097/RLI.0b013e3182588263 22627945 10.1097/RLI.0b013e3182588263

[20] Geiger T Towbin H Cosenti-Vargas A, et al. Neutralization of interleukin-1 beta activity in vivo with a monoclonal antibody alleviates collagen-induced arthritis in DBA/1 mice and prevents the associated acute-phase response. Clin Exp Rheumatol. 1993;11(5):515–522. 8275587

[21] Awan Z Denis M Roubtsova A, et al. Reducing vascular calcification by anti-IL-1β monoclonal antibody in a mouse model of familial hypercholesterolemia. Angiology. 2016;67(2):157–167. doi:10.1177/0003319715583205 25904765 10.1177/0003319715583205

[22] Ridker PM Everett BM Thuren T, et al. Antiinflammatory therapy with canakinumab for atherosclerotic disease. N Engl J Med. 2017;377(12):1119–1131. doi:10.1056/NEJMoa1707914 28845751 10.1056/NEJMoa1707914

[23] Makowski MR Wiethoff AJ Blume U, et al. Assessment of atherosclerotic plaque burden with an elastin-specific magnetic resonance contrast agent. Nat Med. 2011;17(3):383–388. doi:10.1038/nm.2310 21336283 10.1038/nm.2310

[24] Onthank D Yalamanchili P Cesati R, et al. Abstract 1914: BMS753951: A novel low molecular weight magnetic resonance contrast agent selective for arterial wall imaging. Circulation. 2007;116:II_411–II_412 doi:10.1161/circ.116.suppl_16.II_411-c

[25] von Bary C Makowski M Preissel A, et al. MRI of coronary wall remodeling in a swine model of coronary injury using an elastin-binding contrast agent. Circ Cardiovasc Imaging. 2011;4(2):147–155. doi:10.1161/CIRCIMAGING.109.895607 21378029 10.1161/CIRCIMAGING.109.895607

[26] Johnston WF Salmon M Pope NH, et al. Inhibition of interleukin-1β decreases aneurysm formation and progression in a novel model of thoracic aortic aneurysms. Circulation. 2014;130(11 Suppl 1):S51–S59. doi:10.1161/CIRCULATIONAHA.113.006800 25200056 10.1161/CIRCULATIONAHA.113.006800 PMC5097450

[27] Newman KM Jean-Claude J Li H, et al. Cytokines that activate proteolysis are increased in abdominal aortic aneurysms. Circulation. 1994;90(5 Pt 2): II224–227. 7955258

[28] Abdul-Hussien H Hanemaaijer R Kleemann R, et al. The pathophysiology of abdominal aortic aneurysm growth: corresponding and discordant inflammatory and proteolytic processes in abdominal aortic and popliteal artery aneurysms. J Vasc Surg. 2010;51(6):1479–1487. doi:10.1016/j.jvs.2010.01.057 20488324 10.1016/j.jvs.2010.01.057

[29] Pyo R Lee JK Shipley JM, et al. Targeted gene disruption of matrix metalloproteinase-9 (gelatinase B) suppresses development of experimental abdominal aortic aneurysms. J Clin Invest. 2000;105(11):1641–1649. doi:10.1172/JCI8931 10841523 10.1172/JCI8931 PMC300851

[30] Daugherty A Cassis LA Mechanisms of abdominal aortic aneurysm formation. Curr Atheroscler Rep. 2002;4(3):222–227. doi:10.1007/s11883-002-0023-5 11931720 10.1007/s11883-002-0023-5

[31] Dinarello CA Interleukin-1 in the pathogenesis and treatment of inflammatory diseases. Blood. 2011;117(14):3720–3732. doi:10.1182/blood-2010-07-273417 21304099 10.1182/blood-2010-07-273417 PMC3083294

[32] Hashimoto Y Fukazawa R Nagi-Miura N, et al. Interleukin-1beta inhibition attenuates vasculitis in a mouse model of Kawasaki disease. J Nippon Med Sch. 2019;86(2):108–116. doi:10.1272/jnms.JNMS.2019_86-206 31130561 10.1272/jnms.JNMS.2019_86-206

[33] Kitamura K Isoda K Akita K, et al. Abstract 11695: An anti-interleukin-1β antibody suppresses both angiotensin II-induced renal inflammation and hypertension. Circulation. 2017;136:A11695–A11695. doi:10.1161/circ.136.suppl_1.11695

[34] Golledge J Tsao PS Dalman RL, et al. Circulating markers of abdominal aortic aneurysm presence and progression. Circulation. 2008;118(23):2382–2392. doi:10.1161/CIRCULATIONAHA.108.802074 19047592 10.1161/CIRCULATIONAHA.108.802074 PMC2752737

[35] Hong H Yang Y Liu B, et al. Imaging of abdominal aortic aneurysm: the present and the future. Curr Vasc Pharmacol. 2010;8(6):808–819. doi:10.2174/157016110793563898. 20180767 10.2174/157016110793563898 PMC2891873

[36] Okamura H Pisani LJ Dalal AR, et al. Assessment of elastin deficit in a Marfan mouse aneurysm model using an elastin-specific magnetic resonance imaging contrast agent. Circ Cardiovasc Imaging 2014;7(4):690–696. doi:10.1161/CIRCIMAGING.114.001658 24814820 10.1161/CIRCIMAGING.114.001658

[37] Rooke TW Hirsch AT Misra S, et al. 2011ACCF/AHA focused update of the guideline for the management of patients with peripheral artery disease (updating the 2005 guideline): a report of the American College of Cardiology Foundation/American Heart Association Task Force on practice guidelines. J Am Coll Cardiol. 2011;58(19):2020–2045. doi:10.1016/j.jacc.2011.08.023 21963765 10.1016/j.jacc.2011.08.023 PMC4714326

[38] Sakalihasan N Limet R Defawe OD Abdominal aortic aneurysm. Lancet. 2005;365(9470):1577–1589. doi:10.1016/S0140-6736(05)66459-8 15866312 10.1016/S0140-6736(05)66459-8

[39] Ridker PM Howard CP Walter V, et al. Effects of interleukin-1beta inhibition with canakinumab on hemoglobin A1c, lipids, C-reactive protein, interleukin-6, and fibrinogen: a phase IIb randomized, placebo-controlled trial. Circulation. 2012;126(23):2739–2748. doi:10.1161/CIRCULATIONAHA.112.122556 23129601 10.1161/CIRCULATIONAHA.112.122556

[40] Daugherty A Manning MW Cassis LA Angiotensin II promotes atherosclerotic lesions and aneurysms in apolipoprotein E-deficient mice. J Clin Invest. 2000;105(11):1605–1612. 2000/06/07 doi:10.1172/JCI7818 10841519 10.1172/JCI7818 PMC300846

[41] Daugherty A Cassis LA Mouse models of abdominal aortic aneurysms. Arterioscler Thromb Vasc Biol. 2004;24(3):429–434. doi:10.1161/01.ATV.0000118013.72016.ea 14739119 10.1161/01.ATV.0000118013.72016.ea

[42] Cassis LA M Thayer S, et al. ANG II infusion promotes abdominal aortic aneurysms independent of increased blood pressure in hypercholesterolemic mice. Am J Physiol Heart Circ Physiol. 2009;296(5):H1660–1665. doi:10.1152/ajpheart.00028.2009 19252100 10.1152/ajpheart.00028.2009 PMC2685354

[43] ongo GM Xiong W Greiner TC, et al. Matrix metalloproteinases 2 and 9 work in concert to produce aortic aneurysms. J Clin Invest. 2002;110(5):25–632. doi:10.1172/JCI15334 10.1172/JCI15334 PMC151106 12208863

[44] Ishibashi M Egashira K Zhao Q, et al. Bone marrow-derived monocyte chemoattractant protein-1 receptor CCR2 is critical in angiotensin II-induced acceleration of atherosclerosis and aneurysm formation in hypercholesterolemic mice. Arterioscler Thromb Vasc Biol. 2004;24(11):e174–178. doi:10.1161/01.ATV.0000143384.69170.2d 15331433 10.1161/01.ATV.0000143384.69170.2d

